# Academic student satisfaction and perceived performance in the e-learning environment during the COVID-19 pandemic: Evidence across ten countries

**DOI:** 10.1371/journal.pone.0258807

**Published:** 2021-10-20

**Authors:** Damijana Keržič, Jogymol Kalariparampil Alex, Roxana Pamela Balbontín Alvarado, Denilson da Silva Bezerra, Maria Cheraghi, Beata Dobrowolska, Adeniyi Francis Fagbamigbe, MoezAlIslam Ezzat Faris, Thais França, Belinka González-Fernández, Luz Maria Gonzalez-Robledo, Fany Inasius, Sujita Kumar Kar, Kornélia Lazányi, Florin Lazăr, Juan Daniel Machin-Mastromatteo, João Marôco, Bertil Pires Marques, Oliva Mejía-Rodríguez, Silvia Mariela Méndez Prado, Alpana Mishra, Cristina Mollica, Silvana Guadalupe Navarro Jiménez, Alka Obadić, Daniela Raccanello, Md Mamun Ur Rashid, Dejan Ravšelj, Nina Tomaževič, Chinaza Uleanya, Lan Umek, Giada Vicentini, Özlem Yorulmaz, Ana-Maria Zamfir, Aleksander Aristovnik

**Affiliations:** 1 Faculty of Public Administration, University of Ljubljana, Ljubljana, Slovenia; 2 Faculty of Social Sciences, University of Ljubljana, Ljubljana, Slovenia; 3 Faculty of Educational Sciences, Walter Sisulu University, Mthatha, South Africa; 4 Faculty of Education and Humanities, University of Bío Bío, Concepción, Chile; 5 Department of Oceanography and Limnology, Federal University of Maranhão, São Luís, Brazil; 6 Social Determinant of Health Research Center, Department of Public Health, School of Health, Ahvaz Jundishapur University of Medical Sciences, Ahvaz, Iran; 7 Faculty of Health Sciences, Medical University of Lublin, Lublin, Poland; 8 Department of Epidemiology and Medical Statistics, Faculty of Public Health, College of Medicine, University of Ibadan, Ibadan, Nigeria; 9 Department of Clinical Nutrition and Dietetics, College of Health Sciences, University of Sharjah, Sharjah, United Arab Emirates; 10 Centre for Research and Studies in Sociology, Cies-Iscte, Portugal; 11 Department of Sciences and Engineering, Universidad Iberoamericana Puebla/Red Citeg, Mexico City, Mexico; 12 Facultad de Medicina, Universidad Autónoma del Estado de Morelos, Morelos, Mexico; 13 Faculty of Economic and Communication, Bina Nusantara University, West Jakarta, Indonesia; 14 Department of Psychiatry, King George’s Medical University, Lucknow, India; 15 John von Neumann Faculty of Informatics, Obuda University, Budapest, Hungary; 16 Faculty of Sociology and Social Work, University of Bucharest, Bucharest, Romania; 17 Faculty of Philosophy and Letters, Universidad Autónoma de Chihuahua, Chihuahua, Mexico; 18 William James Centre for Research, ISPA—Instituto Universitário, Lisbon, Portugal; 19 Higher Institute of Engineering of Porto, Polytechnic Institute of Porto, Porto, Portugal; 20 División de Investigación Clínica, Centro de Investigación Biomédica de Michoacán, Instituto Mexicano del Seguro Social, Mexico, Mexico; 21 Faculty of Social Sciences and Humanities, ESPOL Polytechnic University, Guayaquil, Ecuador; 22 Faculty of Community Medicine, KIMS, Bhubaneswar, KIIT University, Bhubaneswar, India; 23 Department of Statistical Sciences, Sapienza University of Rome, Rome, Italy; 24 DTI-CUCEA & Instituto de Astronomía y Meteorología—CUCEI, Universidad de Guadalajara, Guadalajara, Mexico; 25 Faculty of Economics and Business, University of Zagreb, Zagreb, Croatia; 26 Department of Human Sciences, University of Verona, Verona, Italy; 27 Department of Agricultural Extension and Rural Development, Patuakhali Science and Technology University, Barisal, Bangladesh; 28 Business Management, University of South Africa (UNISA), Pretoria, South Africa; 29 Department of Econometrics, Faculty of Economics, University of Istanbul, Istanbul, Turkey; 30 National Scientific Research Institute for Labour and Social Protection, Bucharest, Romania; Univerza v Mariboru, SLOVENIA

## Abstract

The outbreak of the COVID-19 pandemic has dramatically shaped higher education and seen the distinct rise of e-learning as a compulsory element of the modern educational landscape. Accordingly, this study highlights the factors which have influenced how students perceive their academic performance during this emergency changeover to e-learning. The empirical analysis is performed on a sample of 10,092 higher education students from 10 countries across 4 continents during the pandemic’s first wave through an online survey. A structural equation model revealed the quality of e-learning was mainly derived from service quality, the teacher’s active role in the process of online education, and the overall system quality, while the students’ digital competencies and online interactions with their colleagues and teachers were considered to be slightly less important factors. The impact of e-learning quality on the students’ performance was strongly mediated by their satisfaction with e-learning. In general, the model gave quite consistent results across countries, gender, study fields, and levels of study. The findings provide a basis for policy recommendations to support decision-makers incorporate e-learning issues in the current and any new similar circumstances.

## Introduction

COVID-19, as a global public health crisis, has been brutal on the economy, education and food security of people all around the world, regardless of national boundaries. Affected sectors include tertiary education, featuring one of the worst disruptions during the lockdown periods given that most countries have tried to keep their essential economic activities running. Still, such activities did not extend to higher education institutions (HEIs), which were closed completely after the suspension of face-to-face activities in an effort to avoid the virus spreading among their students and staff and, in turn, the general population.

Nevertheless, HEIs have continued to offer education by using various digital media, e-learning platforms and video conferencing systems. The result is that e-learning has become a compulsory educational process. Many HEIs were even encountering this mode of delivery for the first time, making the transition particularly demanding for them since no time was available to organize and adapt to the new landscape for education. Both teachers and students today find themselves in a new environment, where some seem better at adapting than others. This means the quality of teaching and learning call for special consideration. In this article, the term “e-learning” refers to all forms of delivery for teaching and learning purposes that rely on different information communication technologies (ICTs) during the COVID-19 lockdown.

To understand COVID-19’s impact on the academic sphere, especially on students’ learning effectiveness, we explored the factors influencing how students have perceived their academic performance since HEIs cancelled their onsite classes. Students’ satisfaction in e-learning environments has been studied ever since the new mode of delivery via ICT first appeared (e.g. [1]), with researchers having tried to reveal factors that shape success with the implementation of e-learning systems (e.g. [2–4]), yet hitherto little attention has been paid to this topic in the current pandemic context. This study thus aims to fill this gap by investigating students’ e-learning experience in this emergency shift. Therefore, the questions we address in the paper are:

R1: Which factors have contributed to students’ greater satisfaction with the e-learning during the COVID-19 pandemic?
R2: Are there any differences between factors influencing quality of the e-learning regarding countries, gender, and fields of study?
R3: How does the students’ satisfaction with the transition to e-learning during the COVID-19 pandemic relate to their academic performance?

According to previous research and considering the new circumstances (e.g. [5–7]), we propose a model for explaining students’ perceived academic performance. In order to identify relevant variables positively affecting students’ performance, we use data from the multi-country research study “Impacts of the COVID-19 Pandemic on Life of Higher Education Students”, coordinated by the Faculty of Public Administration, University of Ljubljana, Slovenia [8]. Structural equation modelling (SEM) is applied to explore the causal relationships among latent concepts, measured by several observed items. Since the SEM approach has a long history of successful applications in research, especially in the social sciences [9, 10] and also in the educational context [11], it offers a suitable statistical framework that allows us to define a conceptual model containing interrelated variables connected to e-learning’s effect on students’ performance [9, 10].

This study significantly contributes to understanding of students’ satisfaction and performance in the online environment. The research findings may be of interest to higher education planners, teachers, support services and students all around the world.

## E-learning and the COVID-19 pandemic

According to the International Association of Universities (IAU), over 1.5 billion students and young people around the globe have been affected by the suspension of school and university classes due to the pandemic [12]. Thus, to maintain continuity in learning while working on containing the pandemic, countries have had to rely hugely on the e-learning modality, which may be defined as learning experiences with the assistance of online technologies. However, most HEIs were unprepared to effectively deal with the abrupt switch from on-site classes to on-line platforms, either due to infrastructure unavailability or the lack of suitable pedagogic projects [13, 14]. To understand the mechanism and depth of the effects of COVID-19, many research studies have been carried out across the world.

Before COVID-19, as new technologies were developed, different e-learning modalities like blended learning and massive open online courses were gradually spreading around the world during the last few decades [15, 16]. Hence, e-learning was deeply rooted in adequate planning and instructional design based on the available theories and models. It should be noted at the outset that what has been installed at many HEIs during the pandemic cannot even be considered e-learning, but emergency remote teaching, which is not necessarily as efficient and effective as a well-established and strategically organized system [17]. Still, all over the world online platforms, for example MS Teams, Moodle, Google Classroom, and Blackboard are in use. Although e-learning offers some educational continuity when it comes to academic learning, technical education has suffered doubly since the social distancing requirements have disrupted the implementation of both practical and work-based learning activities, which are critical for educational success [18].

According to Puljak et al. [19], while students have mostly been satisfied with how they have adapted to e-learning, they have missed the lectures and personal communication with their teachers. They declared that e-learning could not replace regular learning experiences; only 18.9% of students were interested in e-learning exclusively in the long run. Inadequate readiness among teachers and students to abruptly switch from face-to-face teaching to a digital platform has been reported [20].

The closure of universities and schools due to the COVID-19 pandemic has led to several adverse consequences for students, such as interrupted learning, giving students fewer opportunities to grow and develop [21]. This shift has resulted in various psychological changes among both students and teachers [22] and greatly affected their performance. Tutoring system in higher education is an established model of support, advice, and guidance for students in higher education with a purpose to improve motivation and success and prevent drop-out. Pérez-Jorge et al. [23] studied the effectiveness of the university tutoring system during the Covid-19 pandemic. The relation between tutor and student is based on collaboration and communication, which required to adopting quickly to the new situations using different communication technology. The research focused on four different forms of tutoring: in person, by e-mail, using virtual tutoring (Hangout/Google Meet) and WhatsApp. They pointed out that synchronous models and frequent daily communication are essential for effective and successful tutoring system where application WhatsApp, with synchronous communication by messages and video calls, is the form with which students were most satisfied and gain the most from it.

The goal of shifting teaching and learning over to online platforms is to minimize in-person interactions to reduce the risk of acquiring COVID-19 through physical contact. The form of interaction has also moved from offline mode to online mode. Students interact with each other in online platforms for their close group and also for larger groups [24, 25]. Many clinical skills are learned through direct interactions with patients and caregivers, one area that has been badly affected by the switch to e-learning platforms [26–28].

## Student satisfaction with e-learning

Student satisfaction has been shown to be a reliable proxy for measuring the success of implementing ICT-based initiatives in e-learning environments. Scholars have documented a strong relationship between how students perceive their academic performance and how satisfied students are with their e-learning environments [1, 29–31].

The literature reveals important antecedents related to students’ satisfaction with e-learning training, such as online interactions [32, 33], computer efficiency [34, 35], online skills [36], teacher support [34, 37, 38], course design [29, 39], teacher feedback [40], quality of information and activity [1] and technical support [34, 36, 41]. During the COVID-19 pandemic, environmental aspects like temperature, lighting and noise have been identified as significant determinants of students’ e-learning performance [42].

Sun et al. [1] consider the effect of overall quality–as a holistic construct–on satisfaction with the e-learning system. Their research identifies several quality factors that facilitate e-learning through factors associated with: learners (mental health, self-efficacy and attitude of the learner), teachers/instructors (attitude and response timelines assigned by the teacher), technology (quality of technology and the Internet), curriculum (quality and flexibility of the curriculum), design (usefulness and complexity of the design) and environment (interactiveness and assessment diversity). This pandemic has challenged HEIs around the world since e-learning requires physical equipment such as computers, servers, learning and communication platforms, but also software applications, operating systems and experts in the use of these technologies. However, teachers must also possess sufficient digital competencies if they are to use ICT effectively in the learning process.

One of the most relevant factors related to success in implementing e-learning relates to how online education is conducted [19]. This includes receiving timely feedback, teachers’ efforts to be organized, delivering online lectures (and recording them), adapting instructions to this learning model, and helping students follow the courses and look for feedback on their experiences. In some cases, students have not been appropriately guided to follow their courses, overloaded with too many assignments, while there has been a general concern about the lack or loss of practical instruction, which has thus not entirely been covered in their e-learning experiences.

According to Chopra et al. [37], timely feedback and responses to students’ actions are key to effective online delivery. Another study also found a positive association between e-service and information quality with students’ satisfaction [43]. Based on interviews with teachers and students from Jordan, Almaiah et al. [44] found that it is crucial to analyse students and teachers’ use and adoption of systems, while their critical challenges included: (1) change management, students’ and teachers’ resistance, since many prefer traditional learning; (2) ICT literacy; (3) students’ self-efficacy and motivation; and (4) technical issues around systems’ accessibility, availability, usability, reliability, personalization, and service quality, mainly because perceived ease of use might benefit students’ performance and their efficacy while using e-learning systems. Perceived ease of use influences both system adoption and perceived usefulness and was clearly an important aspect since many participants complained that the e-learning system implemented was neither easy to use nor flexible, and this affected their experience regarding technical issues.

An Indian study reports a decline in teacher–student interaction when teaching moved across to online platforms [22]. Hence, greater autonomy is required from students, along with self-regulation and skills to learn online for effective learning [45].

Yet, students’ expertise in computer use and different learning platforms deeply influences their participation in e-learning [34]. Similarly, Wu et al. [35] emphasize the lack of adequate computer skills as an important impediment to effective online delivery. It is important to note that not only the lack of soft skills but also not having adequate hardware can obstruct e-learning. The Hungarian Rectors’ Conference [46], on the basis of 42 Hungarian HEIs’ responses, reported that the experiences with e-learning were generally positive. Still, the main issues involved the lack of technical preparation and equipment; in particular, many students did not have adequate equipment or Internet access. The levels of the students’ satisfaction with the e-learning was also reported to be better among students in developed countries than their counterparts in developing ones [26]. Similarly, resource-scarce settings struggle with the unavailability of digital platforms for education, limited Internet access, poor Internet speed, high cost of Internet and inadequate expertise to work via digital platforms [14]. The infrastructure resources in developing countries are incomparable to developed ones because there is a lack of technological infrastructure for e-learning like computers, connectivity and electricity on top of deficient skills and the active participation of both students and teachers due to insufficient ICT literacy [47].

To strengthen e-learning, the following strategies have been suggested as useful:

- To use a wide variety of learning strategies [48].
- To use tools that allow students to collaboratively build knowledge, discuss, co-construct and interact with the content [49].
- To incorporate social media in e-learning so as to provide an adequate and more engaging learning space [50].
- To use flexible and scaffolded online resources so as to acquire new technical skills that may be useful for future working opportunities [51].
- To provide adequate technological infrastructure and equipment for e-learning [26].

## Students’ satisfaction and performance

Several comprehensive models have also been developed for studying e-learning performance. The technology acceptance model (TAM) provides an easy way to assess the effects of two beliefs–perceived usefulness and perceived ease of use–on users’ intention to utilize a certain technology, hence providing a good prediction of students’ participation and involvement in e-learning, which in turn influences their performance [52].

Rizun and Strzelecki [53] employed an extension of the TAM, which suggests that acceptance of e-learning is related to enjoyment and self-efficacy. According to DeLone and McLean [54], system usage–the degree to which an individual uses the capabilities of a given information system in terms of frequency, nature and duration of use–has a direct connection with users’ satisfaction and their online performance. By applying DeLone and McLean’s Model (D&M model) of Information Systems Success, Aldholay et al. [55] were able to prove that system, service and information quality related to e-learning have significant positive effects on system usage, that thereby predicts a user’s satisfaction and has a positive impact on their performance.

Recently, Al-Fraihat et al. [41] used a multidimensional and comprehensive model and found seven types of quality factors that influence the success of e-learning systems, namely: technical system quality, information quality, service quality, education system quality, support system quality, learner quality, and instructor quality as antecedents of perceived satisfaction, perceived usefulness, use and benefits of e-learning. Moreover, Baber [56] relates students’ perception of their learning outcomes and their satisfaction to factors like students’ motivation, course structure, the instructor’s knowledge and facilitation.

Cidral et al. [34] proposed 11 different constructs of effective e-learning, among which we can mention individual skills, system requirements, and interaction-focused elements. System use and user satisfaction were shown to exert the greatest positive impact on individuals’ performance through e-learning. In a similar study, Hassanzadeh et al. [57] identified the following factors as responsible for success with e-learning: use of the system, loyalty to the system, benefits of using the system, intention to use, technical system quality, service quality, user satisfaction, goal achievement, and content and information quality.

Rashid and Yadav [58] draw attention to several critical issues that may affect the effectiveness of e-learning: students’ possibility to have access to and to afford e-learning technologies; the need for educators to be properly trained in the use of the technologies; teachers’ autonomy and trust; and the quality of the communication among higher education stakeholders. Moreover, Deloitte [59] highlights the importance of institutional support in the successful delivery of e-learning.

## Constructs of the conceptual model and research hypotheses

This study proposes a conceptual model for analysing students’ perceived academic performance during the period of the COVID-19 pandemic, which forced the transition from on-site to on-line teaching and learning. In this research, we combine the theoretical results of previous studies on e-learning with the emergency changeover to various online modes of delivery in response to the pandemic lockdown. The proposed conceptual model builds on the model of students’ satisfaction with e-learning suggested by Sun et al. [1] as well as the D&M model [60], which was used to describe different information systems’ success, including the e-learning system [41]. Cidral et al. [34] studied similar key apects of quality e-learning systems.

In the conceptual model we propose second-order multidimensional construct E-learning Quality of five components. Based on the literature [1, 34, 37] the construct connects three aspects of quality: learner, teacher and system.

Two factors associated with students’ satisfaction corresponding to the learner dimension are included in our proposed model: *Home Infrastructure* and *Computer Skills*. The rapid transition to online study meant students were relocated to a home environment where many did not enjoy suitable conditions to study, both a quiet place and digital equipment with access to (high-performance) Internet, which is indispensable for effective online study. Therefore, the latent variable *Home Infrastructure* covers the ICT conditions at home, i.e. having one’s own computer or access to one, the required software, a webcam, and a stable (and fast) Internet connection [37]. The greater the students’ previous knowledge and experience in using digital media, the easier the transition to e-learning has been. *Computer Skills* describe students’ expertise in using computers and different learning platforms, which is particularly important for active participation in the online delivery mode [34, 35].

The teacher dimension refers to the organization of teaching in a new e-learning environment. Studies show the organization and delivery of study material is important for student satisfaction and performance. Three constructs related to teachers are defined in the model. *Mode of Delivery* corresponds to the different forms used in online lectures, tutorials or practical classes providing learning materials and assignments, such as videoconference, audio recording, forum or e-mail [57]. Teachers play a valuable active role in the online environment by guiding students through the learning contents and providing them with timely responses and information. Equally important are prepared assignments that encourage and motivate students to independently learn at home. *Online Instruction* focuses on teachers’ active role and attitude to online teaching. The construct is explained by *Information Quality* and two other aspects assessed in our questionnaire, namely preparing regular assignments and being open to students’ suggestions [34, 41, 61]. *Information Quality* measures teachers’ responsiveness to the students, such as timely feedback or answering questions in an e-learning environment [34, 37]. We also propose a second-order construct *System Quality*, composed of learner and teacher dimensions: *Home Infrastructure* and *Mode of Delivery*.

Previous studies reveal that IT service support has a positive influence on users’ perceptions of their satisfaction with the system. As the transition to online study happened quickly and without prior training, the support of both the IT and the administrative service is vital for ensuring that students are satisfied with their new learning environment [34, 37, 41, 57, 61, 62]. In our model, *Service Quality* refers to the aspect of administrative, technical and learning assistance. To compensate for the lack of social contact while studying from home, various forms of online interactions are possible. Teacher–student or student–student interactions were shown to be important factors of satisfaction with the e-learning system [34, 41, 61]. The construct *Online Interactions* describes how often a student communicates with colleagues from the course, the teachers or the administrative staff.

To summarize, E-learning Quality is multidimensional construct of five components *Students’ Computer Skills*, *System Quality*, which reflects the *Mode of Delivery* and *Home Infrastructure*,. *Online Instruction* assessed through *Information Quality*, *Online Service Quality and Online Interactions* with colleagues, teachers and staff. We hypothesize:

H1: *Students’ Computer Skills* is correlated with *Home Infrastructure*.

During the COVID-19 pandemic, teaching and learning were completely implemented in the online environment and thus we include the quality dimension, which measures several important aspects of the e-learning system: system quality, information quality, service quality, learner digital quality and interaction quality. Models measuring the success of the information system (also the e-learning system) are usually based on the D&M model, where user satisfaction and quality dimension play an important role [34, 41, 57, 61]. The construct *Perceived Student Satisfaction* is manifested by students’ satisfaction with the organization of e-learning (i.e. lectures, tutorials, seminars, mentorships) and with the support of the teachers and the student counselling service [34, 57]. *Perceived Student Performance* aims to capture students’ benefits of using an e-learning system. It measures students’ opinion of their performance and whether it has worsened with the transition to the online learning mode [34, 41, 57]. The proposed model’s structural part includes three constructs: *E-learning Quality*, *Perceived Student Satisfaction* and *Perceived Student Performance*. We may reasonably assume the quality of the e-learning system has a positive effect on satisfaction with the online education environment system, leading to the system’s greater use and thus to improve the student performance. It is unlikely that one can perform well without use of the system.

This leads to three hypotheses being proposed:

H2: *E-learning Quality* has a positive effect on *Perceived Student Satisfaction*.
H3: *Perceived Student Satisfaction* has a positive effect on *Perceived Student Performance*.
H4: *E-learning Quality* has an indirect (mediated by *Perceived Student Satisfaction*) positive effect on *Perceived Student Performance*.

**Fig 1:**
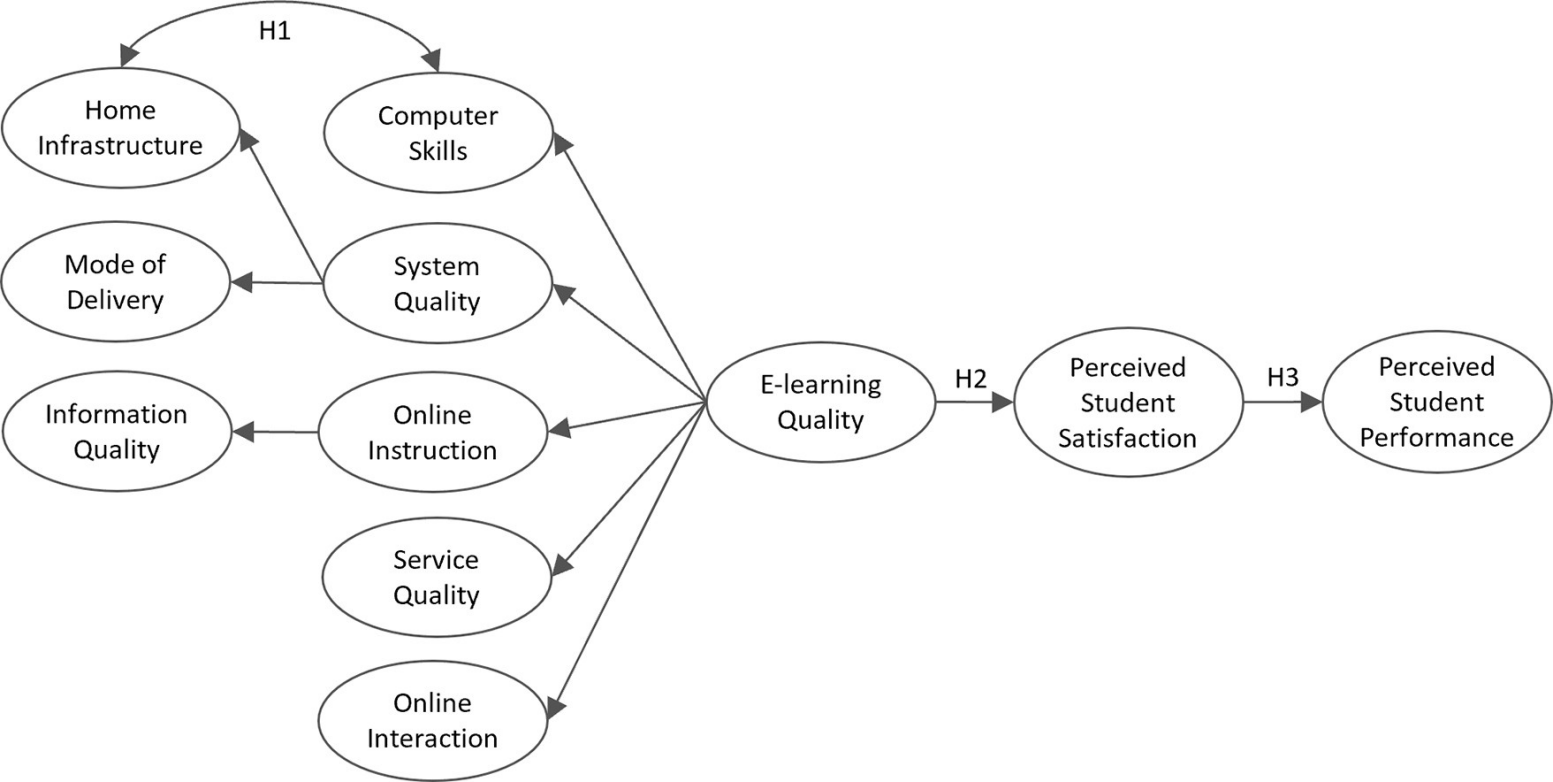
Conceptual model of the perceived student performance in e-learning during the COVID-19 pandemic.

Therefore, we propose the conceptual model presented in Fig 1. and construct description in Table 1.

**Table 1:** Participants’ sociodemographic characteristics per country.

Variable	N	Country									
		Chile	Ecuador	India	Italy	Mexico	Poland	Portugal	Romania	Slovenia	Turkey
		1,137	773	759	1,169	1,368	1,635	863	713	626	1,049
**Student status**	10,040										
Full-time		1,001 (89%)	671 (87%)	738 (98%)	1,101 (94%)	1,121 (82%)	1,508 (92%)	800 (93%)	691 (97%)	612 (98%)	948 (91%)
Part-time		128 (11%)	96 (13%)	14 (1.9%)	65 (5.6%)	240 (18%)	124 (7.6%)	56 (6.5%)	19 (2.7%)	13 (2.1%)	94 (9.0%)
**Level of studies**	9,991										
Bachelor’s		1,094 (97%)	739 (98%)	680 (90%)	706 (61%)	1,250 (92%)	1,012 (62%)	669 (79%)	590 (84%)	528 (85%)	902 (86%)
Master’s		24 (2.1%)	12 (1.6%)	55 (7.3%)	420 (36%)	71 (5.2%)	576 (35%)	129 (15%)	111 (16%)	96 (15%)	110 (11%)
Doctorate		6 (0.5%)	3 (0.4%)	17 (2.3%)	27 (2.3%)	37 (2.7%)	41 (2.5%)	51 (6.0%)	4 (0.6%)	0 (0%)	31 (3.0%)
**Field of studies**	9,968										
Arts and humanities		173 (15%)	60 (7.8%)	4 (0.6%)	162 (14%)	141 (10%)	281 (17%)	84 (9.8%)	37 (5.2%)	26 (4.2%)	209 (20%)
Social sciences		344 (31%)	201 (26%)	13 (1.8%)	599 (51%)	322 (24%)	539 (33%)	293 (34%)	351 (50%)	331 (53%)	699 (68%)
Applied sciences		475 (42%)	273 (35%)	635 (88%)	222 (19%)	457 (34%)	499 (31%)	342 (40%)	203 (29%)	36 (5.8%)	95 (9.3%)
Natural and life sciences		135 (12%)	237 (31%)	66 (9.2%)	182 (16%)	434 (32%)	308 (19%)	135 (16%)	114 (16%)	228 (37%)	23 (2.2%)
**Age** (years)	6,030	24 (4)	24 (3)	21 (2)	23 (3)	24 (3)	22 (3)	26 (12)	24 (2)	21 (2)	23 (3)
**Gender**	10,072										
Male		470 (41%)	353 (46%)	183 (24%)	333 (29%)	446 (33%)	466 (29%)	292 (34%)	142 (20%)	191 (31%)	298 (28%)
Female		643 (57%)	402 (52%)	570 (75%)	828 (71%)	888 (65%)	1,157 (71%)	562 (65%)	566 (80%)	430 (69%)	743 (71%)
Gender diverse		16 (1.4%)	16 (2.1%)	0 (0%)	2 (0.2%)	20 (1.5%)	2 (0.1%)	1 (0.1%)	2 (0.3%)	3 (0.5%)	1 (<0.1%)
Prefer not to say		6 (0.5%)	2 (0.3%)	3 (0.4%)	5 (0.4%)	10 (0.7%)	7 (0.4%)	5 (0.6%)	1 (0.1%)	1 (0.2%)	6 (0.6%)

Note: n (%); except for age—median (interquartile range)

## Materials and methods

### Design and procedure

The data for this study come from a very comprehensive and large-scale global student survey entitled “Impacts of the COVID-19 Pandemic on Life of Higher Education Students”, aimed at examining how students perceive the impacts of the pandemic’s first wave in early 2020 on various aspects of their lives on a global level [8]. This project was originally promoted by the Faculty of Public Administration, University of Ljubljana (Slovenia), which, thanks to the support of international partners, was able to be disseminated worldwide. The online questionnaire was adapted and extended from the European Students’ Union [63] survey. It was formed by 39 questions, mainly including closed-ended questions (see S1 Questionnaire). It focused on socio-demographic, geographic and other aspects pertaining to the life of university students, such as academic online work and life, social life, emotional life, personal circumstances, changes in habits, the roles and measures of institutions, as well as personal reflections on COVID-19 [64]. Initially, the online questionnaire was designed in English and later translated into six different languages (Italian, North Macedonian, Portuguese, Romanian, Spanish, Turkish). The translation of the questionnaire was carried out by native speakers, being proficient in English. The web-based survey was launched via the open-source web application 1KA (One Click Survey; www.1ka.si) on 5 May 2020 and remained open until 15 June 2020, that is, in a period when most nations were experiencing the onerous restrictions imposed by the lockdown. Participation in the study reached global proportions by exceeding the milestone of 30,000 responses submitted by students from more than 130 countries on all six continents. The entire dataset was first analysed by Aristovnik et al. [8].

### Participants

The survey was intended for all higher education students at least 18 years of age, representing the target population of this study. The sampling technique used is non-probabilistic, specifically convenience sampling through university communication systems around the world and social media. The students were informed about the details of the study and gave their informed consent before participating. Due to this study’s specific focus on academic online work and life, it only includes student data with respect to selected parts of the questionnaire. However, since the respondents were not obliged to complete the questionnaire in full, the number of respondents varied across questions. Accordingly, a complete-case-analysis approach was applied to mitigate missing data issues [65]. With the assumption of “missing completely at random”, meaning the complete cases are a random sample of the originally identified set of cases, a complete-case approach is the most common method for handling missing data in many research fields, including educational and epidemiologic research [66, 67]. In order to assure a more robust analysis and perform reliable comparisons on the national level, this study focuses on the 10 countries (Chile, Ecuador, India, Italy, Mexico, Poland, Portugal, Romania, Slovenia, Turkey) that provided at least 500 answers with regard to different aspects of students’ academic life.

The final dataset consisted of 10,092 participants or students enrolled in HEIs, of whom 92% were attending a full-time study course. They were at least 18 years old, with a median age of 23 years (IQR [21.0, 24.0]), and about two-thirds of them (67%) being female. Most respondents (82%) were pursuing a bachelor’s degree, 16% a master’s degree, and 2% a doctoral course. Twelve percent were majoring in a study course in the Arts and Humanities, 37% in the Social Sciences, 32% in the Applied Sciences and 19% in the Natural and Life Sciences. Detailed information on the sample, i.e. the number of respondents and participants’ sociodemographic characteristics by country, are given in Table 1.

### Measures

This study primarily focuses on how COVID-19 has affected different aspects of students’ academic life. Specifically, students reported their experiences with the organization of teaching and administrative services, along with their satisfaction, expectations and perceived impacts on their university career. This involves a total of 34 survey items, representing a basis for measuring the 9 latent constructs used in our proposed conceptual model. Individual satisfaction and concern levels were measured on a 5-point Likert scale, from 1 (lowest value) to 5 (highest value) [68]. A more detailed description, including the set of measuring items and their characteristics, is found in Table 2.

**Table 2:** Items contributing to the definition of the constructs in the conceptual model describing students’ perceived academic performance.

Constructs	Item	Description
Mode of Delivery	Q10b	Satisfaction with video recording of online classes
	Q10c	Satisfaction with audio recording of online classes
	Q12b	Satisfaction with video recording for online tutorials/seminars and practical classes
	Q12c	Satisfaction with audio recording for online tutorials/seminars and practical classes
Home Infrastructure	Q21c	Access to a computer
	Q21d	Required software and programmes
	Q21f	Headphones and microphone
	Q21g	Webcam
Information Quality	Q16b	Feedback on students’ performance regarding given assignments
	Q16c	Replies to students’ questions in a timely manner
	Q16e	Information on what exams will look like in the new situation
Online Instruction	Latent variable	Information Quality
	Q16a	Course assignments (e.g. readings, homework, quizzes) on a regular basis
	Q16d	Feedback to students’ suggestions and adjustments for online classes
Service Quality	Q19b	Satisfaction with technical support or IT services
	Q19c	Satisfaction with student affairs office
	Q19f	Satisfaction with library
	Q19h	Satisfaction with tutors
Online Interaction	Q23f	Interaction with colleagues of my degree course
	Q23g	Interaction with teachers
	Q23h	Interaction with administrative staff
Computer Skills	Q22a	Ability to browse online information
	Q22b	Ability to share digital content
	Q22d	Ability to use online collaboration platforms (Zoom, MS Teams, Skype etc.).
	Q22e	Ability to use online communication platforms (e-mail, messaging etc.)
	Q22f	Ability to use software and programs required for my studies
Perceived Student Satisfaction	Q18a	Satisfaction with online classes
	Q18b	Satisfaction with online tutorials/seminars and practical classes
	Q18c	Satisfaction with online supervisions (mentorships)
	Q19a	Satisfaction with teaching staff
	Q19i	Satisfaction with online student counselling services
Student Perceived Performance	Q20b	Improved performance
	Q20d	Good adaptation to the new teaching and learning experience
	Q20e	Mastery of skills taught in the online classes
	Q20f	Mastery of difficult classwork

### Ethical considerations

All participants were informed about the details of the study and gave their informed consent before participating. By clicking on a button ‘next page’ participants agreed to participate in the survey. Study participation was anonymous and voluntary, and students could withdraw from the study without any consequences. For data-protection reasons, the online survey was open to people aged 18 or over and enrolled in a higher education institution. The procedures of this study comply with the provisions of the Declaration of Helsinki regarding research on human participants. Ethical Committees of several of the higher education institutions involved approved this study, such as the University of Verona (protocol number: 152951), ISPA–Instituto Universitário (Ethical Clearance Number: I/035/05/2020), University of Arkansas (IRB protocol number: 2005267431), Walter Sisulu University (Ethical Clearance Number: REC/ST01/2020) and Fiji National University (CHREC ID: 252.20).

### Data analysis

We implemented the SEM with use of the lavaan package (v.0.6.4, [69]) in the R statistical environment (v.4.0.2, [70]). A two-step approach was followed. In the first step, we checked the fit of the measurement model to all the latent variables; in the second step, we checked the fit of the structural model. The Comparative Fit Index (CFI), Tucker-Lewis Index (TLI), Root Mean Square Error of Approximation (RMSEA) and Square Root Mean Residual (SRMR) were used as goodness of fit indices. The fit was deemed appropriate for CFI and TLI above .90, and for RMSEA and SRMR values below .06 and .08, respectively (e.g. [71, 72]).

We assessed the reliability of the first-order and second-order factors with McDonald’s omega (*ω*) and *ω*_L2_, respectively, and convergent validity with Average Variance Extracted (AVE) using the semTools package (v.0.5.3, [73]). Omega and AVE values above .70 and .50 were indicative of good reliability and convergent validity, respectively [72, 74, 75].

Invariance analysis was performed [72] by comparing the difference in the fit of a series of sequentially constrained models from configural (Conf), intercepts (Intercpy), loadings (Load), means (Means), to regression coefficients (Regr). Invariance was assumed for nonsignificant Δ*χ*^*2*^ or, preferentially, ΔCFI<-.01 for two sequentially constrained models.

## Results

### Preliminary analyses

**Fig 2:**
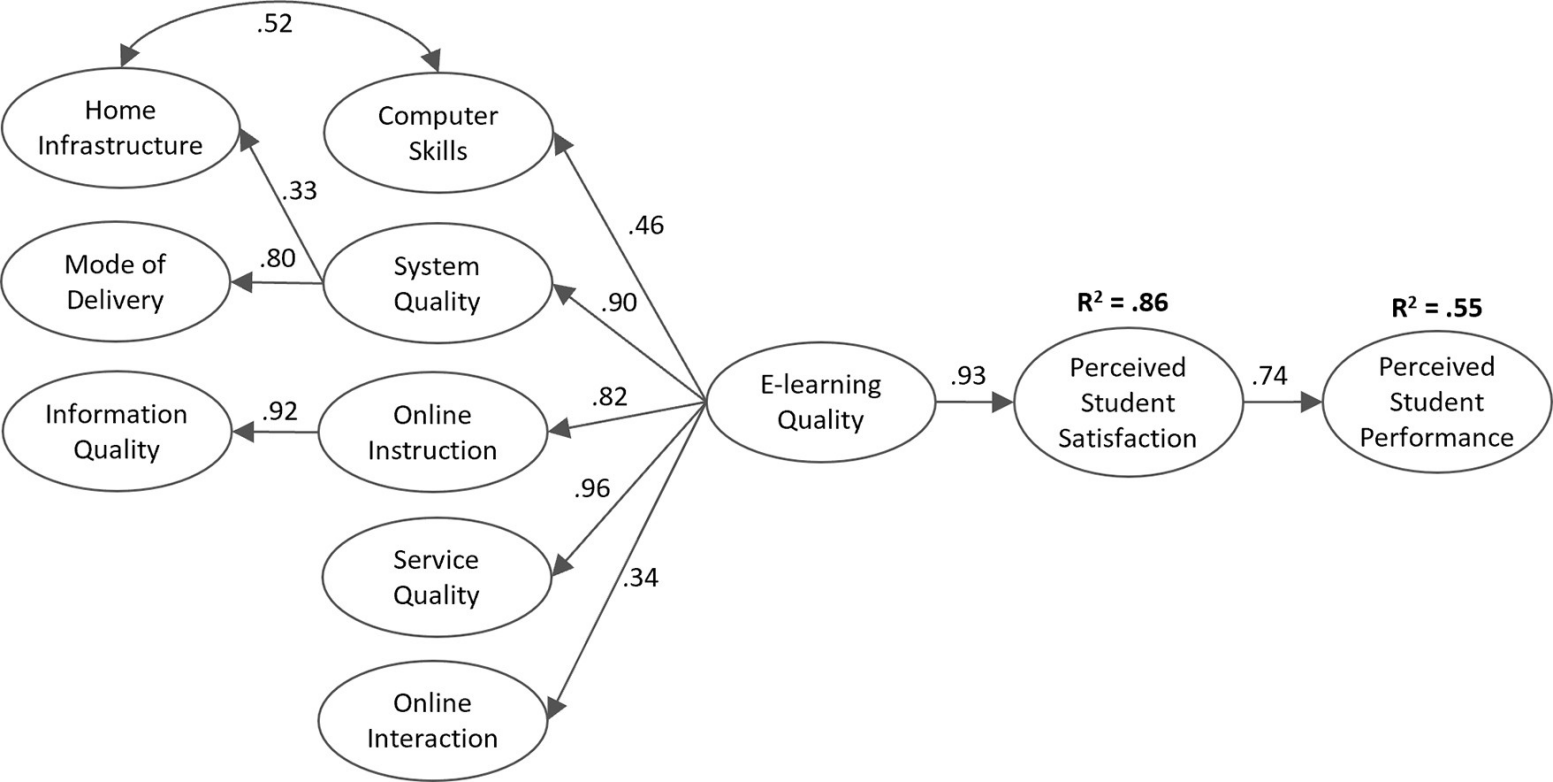
Research model results under the e-learning regime motivated by the COVID-19 pandemic (all path coefficients at p < .001).

Factor loadings and factor reliabilities for the first- and second-order constructs used in the model are given in Table 3. All factor loadings for the first-order constructs were statistically significant for *p* < .001 and larger than the usual .50 cut-off value. Reliability, as measured by McDonald’s *ω*, ranged from .67 (for *Online Instruction*) to .94 (for *Mode of Delivery*). The second-order constructs have lower reliability values, which is explained by the reduced number of indicators in some of these constructs. For the first-order constructs, AVE ranged from .55 (for *Online Interactions*) to .80 (for *Mode of Delivery*). As seen from the reliability measures, the second-order constructs, especially the ones with few indicators, displayed lower AVE. Moreover, in Fig 2, we show the path coefficients calculated for each hypothesis.

**Table 3:** Descriptive statistics (mean, SD), standardized factor loadings (*λ*), standard errors (*SE*), McDonald’s *ω*, and AVE for the latent constructs used in the proposed conceptual model.

Factor	Item	*Mean*	*SD*	*λ*	*SE*	*ω*	AVE
Mode of Delivery	Q10b	3.300	1.141	.890	.006	.920	.803
	Q10c	2.991	1.158	.885	.005		
	Q12b	3.206	1.113	.913	.005		
	Q12c	2.951	1.118	.897	.005		
Home Infrastructure	Q21c	4.465	1.056	.863	.011	.897	.704
	Q21d	4.137	1.126	.897	.009		
	Q21f	4.271	1.189	.775	.014		
	Q21g	4.065	1.396	.815	.012		
Information Quality	Q16b	3.225	1.145	.801	.011	.792	.619
	Q16c	3.619	1.063	.831	.010		
	Q16e	3.499	1.207	.725	.014		
Online Instruction	Information Quality			.919	.002	.665	.550
	Q16a	3.827	1.046	.643	.014		
	Q16d	3.579	1.126	.828	.010		
Service Quality	Q19b	3.178	1.083	.812	.008	.810	.580
	Q19c	3.120	1.073	.790	.009		
	Q19f	3.089	1.097	.616	.014		
	Q19h	3.351	1.021	.807	.009		
Online Interaction	Q23f	3.596	1.583	.661	.023	.723	.551
	Q23g	2.768	1.374	.839	.024		
	Q23h	1.397	0.829	.716	.031		
Computer Skills	Q22a	4.088	0.915	.904	.006	.913	.753
	Q22b	3.981	0.929	.900	.005		
	Q22d	3.939	0.955	.860	.007		
	Q22e	4.256	0.817	.852	.007		
	Q22f	4.088	0.915	.820	.009		
Perceived Student Satisfaction	Q18a	3.384	1.088	.848	.007	.852	.708
	Q18b	3.151	1.122	.857	.007		
	Q18c	3.244	1.069	.843	.007		
	Q19a	3.508	1.039	.840	.008		
	Q19i	3.108	1.024	.818	.008		
Perceived Student Performance	Q20b	2.492	1.119	.689	.015	.845	.580
	Q20e	3.021	1.128	.821	.010		
	Q20d	3.188	1.161	.869	.010		
	Q20f	3.020	1.059	.646	.015		
System Quality	Mode of Delivery			.802	.010	.490	.376
	Home Infrastructure			.329	.024		
E-learning Quality	System Quality			.897	.003	.795	.543
	Online Instruction			.817	.011		
	Service Quality			.958	.006		
	Online Interaction			.338	.025		
	Computer Skills			.463	.017		

Notes: All standardized loadings (*λ*) are statistically significant for *p* < .001. McDonald’s Omega (*ω*) for second-order constructs is *ω*_L2;_ AVE stands for Average Variance Extracted.

### Model of student perceived performance

The overall model under the e-learning regime due to the COVID-19 pandemic is depicted in Fig 2. The estimated model had a good fit to the 10,092 students from the 10 countries that provided more than 500 valid responses (*χ*^*2*^ (519) = 5213.6, *p* < .001, CFI = .990, TLI = .989, RMSEA = .063, SRMR = .049) with all structural paths significant at *p* < .001. The model explained 55% (R^2^ = .55, *p* < .001) of the students’ perceived performance. Major determinants of *E-learning Quality* were *Service Quality* (*β* = .96, *p* < .001) and overall *System Quality* (*β* = .90, *p* < .001). *Online Interactions* with colleagues and teachers (*β* = .34, *p* < .001) and the students’ *Computer Skills* (*β* = .46, *p* < .001) had a lower impact on the e-learning system’s overall quality.

### Country invariance

The analysis of invariance revealed configural invariance (CFI = .900, TLI = .900, RMSEA = .070, SRMR = .060) for the 10 countries. However, no weak measurement invariance (equal loadings between countries) was observed (Δ*χ*^*2*^_Load_ (243) = 510.93, *p* < .001; ΔCFI_Load_ = -.03). Thus, the proposed conceptual model was fit to the 10 participating countries individually. Table 4 summarizes the structural standardized coefficients and fit indices obtained for each country.

**Table 4:** Structural standardized coefficients (*β*) and fit indices by country.

Paths			Country									
			Chile	Ecuador	India	Italy	Mexico	Poland	Portugal	Romania	Slovenia	Turkey
System Qual.	←	E-learning Quality	0.901	0.844	0.853	0.823	0.870	0.892	0.920	0.893	0.857	0.911
Online Instr.	←		0.864	0.864	0.809	0.929	0.878	0.871	0.919	0.847	0.886	0.772
Service Qual.	←		0.868	0.951	0.833	0.786	0.908	0.913	0.736	0.847	0.827	0.904
Online Interact.	←		0.279	0.244	0.768	0.233	0.386	0.263	0.251	0.362	0.197	0.285
Computer Skills	←		0.389	0.474	0.717	0.249	0.459	0.311	0.469	0.250	0.490	0.380
E-learning Quality	→	Perc. Std. Satisf.	0.936	0.911	0.927	0.929	0.924	0.928	0.947	0.953	0.912	0.930
Perc. Std. Satisf.	→	Perc. Std. Perf.	0.696	0.603	0.589	0.608	0.623	0.654	0.767	0.651	0.646	0.626
**Model Fit Indices**												
CFI			0.894	0.894	0.902	0.864	0.903	0.912	0.916	0.902	0.879	0.905
TLI			0.885	0.885	0.894	0.853	0.891	0.905	0.910	0.894	0.870	0.897
RMSEA			0.049	0.050	0.048	0.051	0.047	0.042	0.047	0.046	0.052	0.056
SRMR			0.056	0.054	0.060	0.060	0.050	0.048	0.056	0.061	0.055	0.056
R^2^			0.484	0.363	0.346	0.369	0.388	0.427	0.588	0.424	0.419	0.392

Overall, the models displayed an acceptable fit for all countries (CFI and TLI greater or equal to .850) and for most countries RMSEA and SRMR less or equal than .05 and .06, respectively). The model explained from 35% (India) to 59% (Portugal) of the *Perceived Student Performance* variation within countries. The overall mean explained variance was 42%.

### Gender and areas of study invariance

Invariance analysis of the model revealed strong metric invariance for gender according to the ΔCFI criteria (ΔCFI_Load_ = -.001; ΔCFI_Intercpt_ = -.001), but not for the Δ*χ*^*2*^ criteria (Δ*χ*^*2*^_Load_(26) = 62.253; *p* < .001; Δ*χ*^*2*^_Intercpt_(26) = 79.824; *p* < .001). However, for large sample sizes inflation of *χ*^*2*^ is well known, thus recent research has adopted different criteria, including the ΔCFI as described in the methods section. Using the gender ΔCFI criteria, invariance was also observed for factor means ΔCFI_Means_ <-.001) and structural regression coefficients (ΔCFI_Means_ = -.001).

The model displayed strong metric invariance for the areas of study (Arts and Humanities, Social Sciences, Applied Sciences, Natural Sciences) according to the ΔCFI criteria (ΔCFI_Load_ = -.002; ΔCFI_Intercpt_ = -.003). Using the same criteria, invariance was also observed for factor means ΔCFI_Means_ = -.001) and structural regression coefficients (ΔCFI_Means_ <-.001).

Therefore, we conclude that the model is invariant for gender and areas of study, implying that we can apply it for both genders and all four areas of study.

## Discussion

The goal of this research was to analyse which factors influenced students’ perceived academic performance after switching their academic activities over to the online mode, as imposed by the lockdown in response to COVID-19 in 2020. To this end, a global study including 62 countries was conducted. In this paper, we presented the results of 10 countries that provided more than 500 valid responses.

The study results show that the impact of computer skills is less influential for e-learning quality compared to other factors like system quality, which is the most determinative factor. These results are aligned with previous studies (e.g. [34, 37]), which found that system quality is positively related to a user’s perceived satisfaction, but are contrary to Al-Fraihat et al. [41] who did not detect any significant system quality impact. Our data also show that different modes of delivery positively influenced system quality. On the other hand, even though the quality and diversity of the home infrastructure revealed some impact on the system quality, it is a less determinative factor. These results suggest that students respond better to diversity in learning formats, but it seems that having suitable infrastructure is not so important.

As concerns online instruction, we found that it is one of the three major determinant of e-learning quality and, therefore, for students’ perceived satisfaction and performance. Online instruction can be assessed by the construct information quality, as well as by considering other factors like the teacher’s active role and attitude to online teaching, preparation of regular assignments and openness to the students’ suggestions [34, 41, 61]. Information quality can be explained by teachers’ responsiveness to the students, such as timely feedback or answering questions in an e-learning environment [19, 34, 37].

The active role of teachers and their responsiveness and feedback seem crucial for the students’ satisfaction with the online instruction since the teacher/instructor is a key element of success with the e-learning environment [76]. Sun et al. [1] investigated the instructor’s role in the success of e-learning, focusing on two specific indicators: instructor response timelines, and instructor attitude to e-learning. They found a positive and significant relationship between these aspects and the satisfaction of students. Similar findings were outlined by Cidral et al. [34], who documented a positive relationship between instructor attitude to e-learning and user satisfaction. In addition, Al-Fraihat et al. [41] and Mtebe and Raphael [77] established a positive relationship between the instructor’s quality and students’ perceived satisfaction with an e-learning system. Moreover, the quality of information provided by the instructor/teacher has been considered to be a determinant of perceived satisfaction in previous studies that support our findings [29, 37, 41, 43, 78, 79]. According to Al-Fraihat et al. [41], it is essential to provide students with clear, updated and sufficient information and quality content.

Regarding online service quality, we found that it was a major determinant of the students’ perceived e-learning quality. This allows us to infer that administrative, technical and learning assistance through tutors and the library is very important for students’ greater satisfaction and, in consequence, students’ higher perceived satisfaction and performance. This result is contrary to Cidral et al. [34], yet consistent with the findings of Al-Fraihat et al. [41], Hassanzadeh et al. [57] and Chopra [37], who state that providing quality services might increase the level of satisfaction, making it crucial to have personnel available to support students with their technical issues and satisfy their needs, generating positive feelings towards the e-learning system.

The construct online interactions describes how often a student communicates with colleagues from the course, the teachers or the administrative staff [34, 41]. This factor was considered to be one of the least determinative of overall satisfaction-learning quality and, consequently, least able to explain the conceptual model of perceived student performance. It seems the new emergency remote teaching and learning scenario [17] has affected the frequency of student interactions with colleagues and teachers [19, 22], which may explain why it is less important for perceived e-learning quality. Our results suggest these interactions are still needed for a successful student performance in an e-learning environment, although they are less determinative than other factors.

The first hypothesis (H1) about the influence of the students’ computer skills on e-learning quality and The first hypothesis (H1), referring to the intercorrelation between students’ computer skills with the quality and variety of the IT infrastructure at home, were confirmed. The correlation is only moderate. In other terms, students who possess different digital media and better-quality infrastructure at home had greater digital competencies, which then favoured their perceived e-learning quality and, thus, the students’ perceived satisfaction and performance under the e-learning mode.

Taking all five dimensions of e-learning quality into consideration, the second hypothesis (H2) is also confirmed because this factor (e-learning quality) has a very strong positive effect on perceived student satisfaction. Students who are more satisfied with the quality of their e-learning experience are generally more satisfied with their education, which further more positively influences their perceived academic performance (see H3). Students more satisfied with their online education also perform better at school. The result highlights the role of students’ satisfaction in their academic performance [60]. At the same time, we may infer that students who use the online learning mode more frequently perceive their educational performance is higher.

The last hypothesis (H4) is also confirmed. E-learning quality has an indirect (mediated by perceived student satisfaction) positive effect on perceived student performance. The overarching research question of our study is thereby confirmed: the better the quality of the e-learning system, the more satisfied students are with their academic performances.

Regarding the country comparisons (see Table 2) and considering the overall model’s lack of invariance and irrespective of the country differences, the results show that students’ perceived satisfaction is largely predicted by the quality of the school’s service and the quality of the overall system. However, it is worth discussing some of the outliers shown in Table 2.

Concerning computer skills, one can observe a significant difference between India and the other countries. This result might have been influenced by the fact that the majority of Indian students participating in the study have a technical background, pursuing Engineering or Medical Sciences. Hence, their proficiency in computing is expected to be high. On the other side, among Romanian students the impact of computer skills on e-learning quality is the lowest, which may be explained by the structure of the Romanian sample, comprising more Social Sciences students who prefer face-to-face interactions over the use of different platforms for online teaching, which has increased the workload compared to the previous situation.

While examining the results for the construct system quality, we see that, although most countries show similar structural standardized coefficients, Portugal has a slightly highest coefficient compared to the rest of the countries. This result might be caused by the fact that Portugal had already been through a process of creating a very strong online higher education infrastructure [80], meaning the students’ transition to this modality has been quite smooth and they do not seem to perceive any significant change.

With respect to online interactions, India has a significantly higher coefficient than the corresponding values for the other countries. This result may be explained by the fact that the average university class size in India is 150–250 students, making it very difficult for the students to interact with each other or with the teachers in a personal way. In the new e-learning scenario, teachers are more available for flexible consultation time. In addition, many of the teaching strategies that lecturers are relying on encourage collaborative work. Yet, in contrast, Slovenia has the lowest coefficient for this factor, which can be attributed to the fact that, even before the pandemic outbreak, e-learning was widespread in higher education, including blended learning, and thus the students do not consider that online interactions have increased or changed due to the pandemic.

Regarding online instruction, Turkey has the lowest coefficient of the 10 countries. The high number of Turkish students per academic, which exceeds the OECD average [81] makes it difficult for academics to give individual feedback to all of their students.

Regarding gender and areas of study, the proposed model proved to be invariant for both factors, which confirms its relevance in explaining students’ perceived academic performance through the quality of the e-learning infrastructure as mediated by students’ perceived satisfaction.

Although no significant difference in the results is found by gender, the number of female participants is remarkably higher than for males in all countries. Although the causes of this result lie beyond the scope of this study, it would be worth analysing them in future research.

## Conclusions

Our study has provided insights into latent factors explaining students’ perceived academic performance during the first wave of COVID-19 pandemic, which forced the transition to online education. The results confirmed all of the hypotheses and the proposed conceptual model was revealed to be reliable.

According to the study results, the quality of e-learning during the COVID-19 pandemic’s first wave was mainly derived from service quality with administrative, technical and learning assistance through tutors and the library, teachers’ active role in the process of online education with their responsiveness and timely feedback, and overall system quality with the mode of delivery and IT infrastructure. Students’ digital competencies and online interactions with colleagues and teachers were shown to be slightly less important factors, yet still statistically significant. Moreover, our study shows that the impact of e-learning quality on student performance is strongly mediated by student satisfaction with e-learning.

Understanding the factors that influenced students’ performance after the urgent introduction of e-learning may be important for decision-makers and all those involved in implementation in any future new similar circumstances. Thus, the results of our study imply a clear strategy for education, research and policy. Investment in the development of digital competencies, of both students and academic staff, together with initiatives supporting research and interdisciplinary innovative collaboration within the scope of different aspects of higher online education, are recommended and should be encouraged.

### Limitations

This study has several limitations that should be considered. First, the convenience sampling methodology, which limits the generalizability of the results. The calculated results are based on a sample, which includes students from 10 countries, although European countries prevail. It is clear that the countries are on different levels of economic development and have differently organized and developed higher education systems. Further, no data come from low-income countries, where students might have a problem with an Internet connection and access to appropriate equipment [82, 83]. In addition, to access the online questionnaire students first needed to have electronic devices and an Internet connection, which could cause selection bias.

Another important limitation of this study is the time in which the data were collected. Not all countries were in the same pandemic phase or lockdown period, which might impact the student responses. Therefore, our study does not give a full picture of the students’ perceived satisfaction and performance during e-learning in the time of the first wave of the pandemic.

### Future work

Future research could attempt to cluster countries by their economic development level given that e-learning quality and students’ perceived satisfaction and performance with online education depend on IT technology development and IT tools’ access and affordability [83]. In the future, studies should include representative countries on all levels of development and economic growth to further test the proposed model and look for differences in the area of students’ perceived satisfaction and performance with e-learning. This may help generate evidence for policymakers to invest in developing online education infrastructure in low- and middle-income countries.

Further, although digitalization in HEIs has been confirmed as significant and essential for the higher education system’s functioning during the lockdown [84] and e-learning has offered some kind of continuity of academic education, it does not meet all of the needs for practical and work-based learning, e.g. in medical and health or technical sciences education, especially when viewed in the long run [85–87]. In future research, more emphasis should be placed on analysing students’ perceived satisfaction and performance with online education in the context of differences between fields of study, particularly in relation to the nature of education (theoretical vs. practical) and the competencies that are supposed to be developed during education.

Future research may also consider differences between local and international students’ perceived satisfaction and performance. According to the EMN/OECD report [88], the COVID-19 pandemic has imposed more difficult situations on international students than local students in terms of psychological and financial issues. This may well impact their academic outcomes. Such analysis could also compare the adaptation to the online education environment of students whose training is in their mother language and students for which the training is in a second language.

Finally, the survey is based on the subjective opinion of students, also with regard to their academic performance. Therefore, to objectify the results further research entailing analysis of the relationship between students’ satisfaction with online education and their learning outcomes expressed in the form of grades may reveal interesting results. Namely, recent analyses suggest that students have been receiving higher grades during the pandemic compared to the on-site education before the pandemic, which may increase their satisfaction with the e-education [82].

## Supporting information

S1 Questionnaire (DOCX)

S1 Dataset (XLSX)
