# Supplementary material for: Academic student satisfaction and perceived performance in the e-learning environment during the COVID-19 pandemic: Evidence across ten countries
Source: PLoS One. 2021 Oct 20;16(10):e0258807. doi: 10.1371/journal.pone.0258807 (PMC8528294; doi:10.1371/journal.pone.0258807)
Supplement: S1 Questionnaire — (DOCX) [file pone.0258807.s001.docx]

**Questionnaire 1st Wave – Link**

<http://www.covidsoclab.org/wp-content/uploads/2021/10/Questionnaire-1st-Wave.pdf>
